# *Bacillus multifaciens* sp. nov., a Crucial and Highly-Active Flavor and Protease Producer Isolated from the *qu*-Starter of Chinese *Wuliangye* Baijiu

**DOI:** 10.3390/microorganisms13050993

**Published:** 2025-04-25

**Authors:** Qingchun Luo, Xinrui Zhao, Xi Li, Yuzhu Li, Pengju Zhao, Yanping Lu, Duotao Liu, Jian Su, Jian Chen, Dong Zhao, Jianghua Li, Jia Zheng

**Affiliations:** 1 Science Center for Future Foods, Jiangnan University, 1800 Lihu Road, Wuxi 214122, China; 7220210001@stu.jiangnan.edu.cn (Q.L.); zhaoxinrui@jiangnan.edu.cn (X.Z.); jchen@jiangnan.edu.cn (J.C.); 2 Key Laboratory of Industrial Biotechnology, Ministry of Education, School of Biotechnology, Jiangnan University, 1800 Lihu Road, Wuxi 214122, China; 3 Jiangsu Province Engineering Research Center of Food Synthetic Biotechnology, Jiangnan University, 1800 Lihu Road, Wuxi 214122, China; 4 Engineering Research Center of Ministry of Education on Food Synthetic Biotechnology, Jiangnan University, 1800 Lihu Road, Wuxi 214122, China; 5 Wuliangye Yibin Co., Ltd., Yibin 644000, China; 1710307128@pku.edu.cn (X.L.); liyuzhu@wuliangye.com.cn (Y.L.); zhaopj19@tsinghua.org.cn (P.Z.); luyanpingscut@163.com (Y.L.); ldt1983611@163.com (D.L.); sujian@wuliangye.com.cn (J.S.); zhaodong@wuliangye.com.cn (D.Z.); 6 Solid-State Fermentation Resource Utilization Key Laboratory of Sichuan Province, Yibin 644007, China; 7 Key Laboratory of Wuliangye-Flavor Liquor Solid-State Fermentation, China National Light Industry, Yibin 644000, China

**Keywords:** Chinese baijiu, *Bacillus multifaciens* sp. nov., starter, volatile flavor components, producing protease

## Abstract

In the study presented herein, an aerobic, Gram-stain-positive, spore-forming bacterium, designated as WLY-B-L8^T^, was isolated from a *qu*-starter (*baobaoqu*) cultivation facility used for the production of Wuliangye baijiu in Yibin city (Sichuan province, China). The strain comprised short, rod-shape cells of 1.2–1.9 μm in width and 1.7–4.8 μm in length, arranged singly or in pairs. The isolate was able to grow at temperatures of 20–42 °C (optimum growth at 40 °C), pH 5.0–10.0 (optimum growth at pH 8.0), and in the presence of 0–2% (*w*/*v*) NaCl (optimum growth with 1% NaCl). Ribose, xylose, arabinose, mannose, glucose, and galactose constituted the major cell-wall sugars. Moreover, *meso*-diaminopimelic acid (*meso*-DAP) constituted the diagnostic amino acid. The main polar lipids of WLY-B-L8^T^ included diphosphatidylglycerol (DPG), phosphatidylglycerol (PG), phosphatidylethanolamine (PE), unidentified aminolipids (UAL 1–2), an unidentified aminophospholipid (UAPL), an unidentified aminoglycolipid (UAGL), and an unidentified lipid (UL). MK-7 was the predominant menaquinone and *iso*-C_15:0_ (23.00%) was the major fatty acid. Comparisons of the 16S rRNA gene sequence indicated that WLY-B-L8^T^ was most closely related to *Bacillus rhizoplanae* JJ-63 DSM 12442^T^ (98.71%), *Bacillus pseudomycoides* DSM 12442^T^ (98.21%), and *Bacillus cytotoxicus* NVH 391–98^T^ (98.14%). The average nucleotide identity (ANI) values of strain WLY-B-L8^T^ and the three type strains mentioned above were 88.24%, 80.57%, and 78.70%. The average amino identity (AAI) values between them were 89.84%, 79.51%, and 80.41%. In addition, the digital DNA–DNA hybridization (dDDH) values between them were 36.70%, 26.10%, and 23.90%. The genomic DNA G+C content was 35.97%. Based on the evidence presented herein, WLY-B-L8^T^ (CICC 25210^T^ = JCM 36284^T^) exhibits promise as the type strain of a novel species, designated as *Bacillus multifaciens* sp. nov., that can produce protease (119.38 ± 7.44 U/mL) and volatile flavor components when cultured on raw wheat, such as 2-pipendinone (21.95 ± 1.56 mg/L), phenylethyl alcohol (19.08 ± 0.82 mg/L), hydrocinnamic acid (18.60 ± 0.53 mg/L), and acetoin (7.58 ± 0.11 mg/L).

## 1. Introduction

The genus *Bacillus* holds an important place in the history of bacteriology. It was first reported by Cohn in 1872, with the type species of *Bacillus* being designated as *Bacillus subtilis* [1]. *Bacillus* is a Gram-positive, rod-shaped, aerobic or facultatively anaerobic, endospore-forming bacteria [1,2]. A total of 2789 species have been identified and officially designated thus far (https://lpsn.dsmz.de/genus/Bacillus) (accessed on 18 January 2025) that are phylogenetically and phenotypically heterogeneous, featuring diversity in their morphologies, nutritional requirements, and growth conditions [3]. The most prominent characteristic of members of the genus *Bacillus* is their ability to form endospores to resist adverse conditions that lead to their distribution in a wide range of environments, such as fresh waters, marine sediments, desert sands, hot springs, arctic soils, and the air [4].

Strong-flavored Chinese baijiu is one of the most important flavor-types in China. Its production involves the joint action of various microorganisms from pit mud, *daqu*, and fermented grains [3]. Wuliangye baijiu is fermented using a traditional solid simultaneous saccharification technique dependent on a special fermentation starter (referred to as *daqu* in China) that provides abundant microorganisms, enzymes, and flavor substances [5]. Wuliangye *daqu* (also known as *baobaoqu*) is obtained from pure wheat through solid-state fermentation and is one of the most crucial factors affecting the flavor of baijiu [6]. The manufacturing of *baobaoqu* is a process of directed screening and enriching of functional microorganisms [7]. The findings of an increasing number of studies have shown that *Bacillus* plays an important role in the brewing process of baijiu. The metabolites of *Bacillus* can promote the formation of Baijiu flavor [8,9]. The results of a number of studies have shown that *Bacillus* species, such as *Bacillus licheniformis* and *Bacillus velezensis*, can secrete esterases to synthesize ethyl esters from short-chain fatty acids. Such enzymatic activity increases the concentration of esters, including ethyl acetate, ethyl hexanoate, and ethyl butyrate, in baijiu [10].

In order to study the microbial characteristics of the Wuliangye baijiu environment and the function of microbial production of baijiu flavor components [11], herein, we report on the isolation and characterization of a strictly aerobic, Gram-stain-positive bacterium, designated WLY-B-L8^T^, isolated from the air at a *baobaoqu* cultivation plant for the production of Wuliangye baijiu.

## 2. Materials and Methods

### 2.1. Chemicals and Reagents

Tryptone, yeast extract powder, glucose, NaCl, Bacto agar, NaH_2_PO_4_, and Na_2_HPO_4_ were purchased from Beijing Aobox Bio-tech Co., Ltd. (Beijing, China). Pancreatic cheese peptone and soybean papain hydrolysate were purchased from Huankai Microbial Technology Co., Ltd. (Guangdong, China). 4-octanol was purchased from Sigma-Aldrich Shanghai Trading Co., Ltd. (Shanghai, China, Purity > 98%).

### 2.2. Culture Media

The ingredients used in the Plate Count Agar (PCA) medium are as follows (per liter): 5 g tryptone, 2.5 g yeast extract powder, and 1 g of glucose. The medium was adjusted to pH 7.0. The Tryptose Soya Agar (TSA) medium contained the following constituents (per liter): 15 g of pancreatic cheese peptone, 5 g of soybean papain hydrolysate, and 5 g of NaCl. The medium was adjusted to pH 7.0. The plate media were prepared by adding 15 g/L of Bacto agar. All media were autoclaved at 121 °C and 15 psi for 20 min (HVN-85, Hirayama, Tokyo, Japan).

### 2.3. Isolation and Ecology

WLY-B-L8^T^ was isolated from the air at the Wuliangye 502# *baobaoqu* cultivation facility in Yibin (Sichuan province, China; 28°47′ N, 105°36′ E). Air samples from the 502# *baobaoqu* cultivation facility were screened for bacteria using the Plate Count Agar (PCA) medium. A plankton sampler (FKC-I type, Zhejiang Sujing Purification Equipment Co., Ltd., Shaoxing, China) was used to collect microorganisms in the air for a period of 1 min (total air volume 100 L), with sampling repeated three times. The plates were incubated at 37 °C. The strain was picked as a single colony and further purified through transfer to PCA agar plates several times. The purified strain was stored at −80 °C in tubes containing 20% glycerol solution. The strain was designated as WLY-B-L8^T^.

### 2.4. Physiology and Chemotaxonomy

Cell morphology was observed under a light microscope (BH-2, Olympus, Tokyo, Japan) and through scanning electron microscopy (SEM) after incubation on PCA plates for 72 h at 37 °C [12]. The SEM procedure was as follows: An appropriate amount of bacteria were scraped from the solid medium, fixed with 2.5% glutaraldehyde for 4 h, and then washed with 0.2M PBS buffer (pH 6.8) 3 times for 15–20 min on each occasion. Thereafter, water treatment was performed by applying different concentrations of ethanol (30, 50, 70, 85, 95, or 100%, with each treatment applied once) for 15–20 min on each occasion. The samples were then dried using the critical point of carbon dioxide, sprayed with gold using an ion sputtering instrument, and then observed and photographed with a scanning electron microscope (SU 8010, Hitachi, Tokyo, Japan). Gram staining was performed in solid media at 37 °C, following the method described in a previous study [13]. In order to determine the optimal growth conditions, cultures of WLY-B-L8^T^ were inoculated in PCA for 3 days at 4, 10, 15, 20, 25, 30, 37, 40, 42, 45, and 50 °C. The pH and NaCl (0–5%) ranges for growth were measured at 37 °C and adjusted with NaH_2_PO_4_/Na_2_HPO_4_ buffer to pH 3.0–12.0 at 1.0 pH unit intervals. Growth at different temperatures, pH, and NaCl concentrations was investigated after 72 h.

Acid production from carbon sources was tested using API 50CH strips based on the manufacturer’s instructions (BioMérieux, Lyon, France). Enzyme activity was characterized using API ZYM strips (BioMérieux, Lyon, France). API 20E and API 20NE were used to perform physiological and biochemical tests, including carbon substrate utilization, nitrate reduction, *β*-galactosidase, arginine dihydrolase, citrate utilization, H_2_S production, the Voges–Proskauer test, and the hydrolysis of gelatin, aesculin, and urea. These tests were performed at 37 °C and the 72 h time point. Motilities were tested using the hanging-drop technique with a semi-solid TSA medium containing 0.5% agar. Oxidase activity was determined based on reagent color change using API 20NE kits (BioMérieux, France) and catalase activity was determined based on bubble production in 3% (*v*/*v*) H_2_O_2_.

The cells were grown on TSA at 30 °C for 72 h for fatty acid methyl ester (FAME) identification. Cellular fatty acids were extracted, methylated, and analyzed using the Sherlock Microbial Identification System (MIDI, Palo Alto, CA, USA) based on the manufacturer’s instructions.

### 2.5. Phylogenetic and Genomic Analyses

Genomic DNA of WLY-B-L8^T^ was extracted and purified using the method described by Lane [14]. The 16S rRNA gene sequence was amplified using the universal primers 27F (5′-AGAGTT TGATCCTGGCTCAG-3′) and 1492R (5′-GGTTACCTTGTTACGACTT-3′) [15]. The PCR product was inserted into a pEASY-T1 Cloning Vector and sequenced using the M13 forward primer and M13 reverse primer [3]. A phylogenetic tree based on the 16S rRNA gene sequences from strain WLY-B-L8^T^ and closely related species, obtained from the EzTaxon server 2.1 [16], was reconstructed using the neighbor-joining method [17] in MEGA version 7.0 [18] with bootstrap analysis of 1000 replications.

To support the results of the phylogenetic analysis and to obtain deeper taxonomic insights, the genome of WLY-B-L8^T^ was sequenced. Genome sequencing of WLY-B-L8^T^ was performed on the BGISEQ-500 platform (Beijing Genomics Institute, Beijing, China) [17] and the G+C content of the DNA was determined from whole sequence data [19]. Sequenced read pre-processing, genome de novo assembly, and gene annotation were carried out using Prodigal version v2.6.3.

Using the Genome-to-Genome Distance Calculation (GGDC) website (https://ggdc.dsmz.de/, accessed on 18 January 2025), genome sequence-based digital DNA–DNA hybridization (dDDH) values were calculated with Formula (2), using the method described by Meier-Kolthoff et al. [20]. The average nucleotide identities (ANIs) between the genome of WLY-B-L8^T^ and the type strains’ genomes were calculated using an online tool (https://www.ezbiocloud.net/tools/ani/, accessed on 18 January 2025) [21]. The average amino identity (AAI) value between them was calculated using CompareM (v 0.0.32, https://github.com/donovan-h-parks/CompareM, accessed on 18 January 2025) [22]. The Glimmer 3.02 software was used to annotate the genome of strain WLY-B-L8^T^ and predict coding sequences. The KEGG (Kyoto Encyclopedia of Genes and Genomes), COG (Cluster of Orthologous Groups) and GO (Gene Ontology) databases were used for functional analysis of the predicted coding genes.

### 2.6. Protease Enzyme Production Analyses

*Bacillus* spp. can secrete abundant enzymes to perform starch hydrolysis and proteolysis and produce metabolites [23]. For quantitative analysis of protease production of the new *Bacillus* strain, WLY-B-L8^T^ was streaked onto a PCA plate and incubated at 30 °C for 3 days to form single colonies. These colonies were then inoculated at the center of a protease assay medium plate. The medium consists of 20 g of skim milk powder mixed thoroughly with 600 mL of tap water and sterilized at 115 °C for 20 min, and 20 g agar mixed thoroughly with 400 mL of tap water and sterilized at 121 °C for 20 min. The skim milk and agar solutions were mixed after sterilization. The plates were incubated at 30 °C for 3 days, with three parallel experiments conducted. We performed a quantitative analysis based on the method described below.

#### 2.6.1. Tyrosine Standard Curve

A tyrosine standard solution was prepared based on a method described in a previous study [24]. A tyrosine standard curve was then constructed based on absorbance at 660 nm, with three parallel setups.

#### 2.6.2. Protease Crude Enzyme Solution

Single colonies of the new *Bacillus* strain WLY-B-L8^T^ were inoculated into a fermentation medium (as referenced in the aforementioned literature) and incubated at 30 °C in a shaker at 160 rpm for 3 days. The fermentation broth was subsequently centrifuged at 10,000 rpm for 10 min, and the supernatant was then collected as the crude enzyme solution.

#### 2.6.3. Determination of Protease Activity

Protease activity was measured according to the methods described in the referenced literature. Protease activity is defined as the amount of enzyme required to hydrolyze casein to produce 1 μg of tyrosine per minute at 40 °C and pH 7.2, equivalent to one unit of protease activity (U/mL). Enzyme activity was calculated by using the standard curve, with three parallel experiments conducted.

### 2.7. Flavor Compound Analyses

Fermentation: The WLY-B-L8^T^ strain was inoculated into a liquid NA medium for activation and cultured to the logarithmic phase until the OD_600_ reached 1.8. Thereafter, 5 mL of the WLY-B-L8^T^ strain cultures were inoculated into a 250 mL Erlenmeyer flask containing 50 g of wheat powder (38% moisture content), which were prepared using the *baobaoqu* production process [6]. The control group received the same amount of sterile water. After incubation at 37 °C for 3 days, 10 g culture were added to a 50 mL clean centrifuge tube with 10 mL of dichloromethane, and were shocked thoroughly for 10 min (QB-210, Kylin-Bell Lab Instruments, Haimen, China) and extracted via sonication (40 KHZ) for 20 min at room temperature (US-15M, Zhongkeyi Co., Ltd, Beijing, China). The 2 mL extracted solution was filtered through a 0.22 μm membrane filter, and 10 μL of the internal standard (400 mg/L 4-octanol) were added to the assay bottle. The flavor compounds were analyzed using headspace solid-phase microextraction (HS-SPME) combined with gas chromatography mass spectrometry (GC-MS) [25].

HS-SPME Analysis: Aroma extraction was performed using a 50/30 µm DVB/CAR/PDMS fiber (Supelco, Inc., Bellefonte, PA, USA) following a method outlined previously [26]. A 2 mL sample was placed in a 20 mL vial (item S126-0020, I-CHEM) and saturated with sodium chloride. The vial was then securely sealed with a Teflon/silicone septum. The sample was equilibrated at 50 °C in a thermostatic bath for 15 min and extracted for 30 min at the same temperature with stirring. Following extraction, the fiber was inserted into the GC injection port at 250 °C to desorb the analyte.

GC-MS Analysis: Semi-quantitative analysis was carried out using an Agilent 7890 GC coupled to an Agilent 5977 mass selective detector (MSD), which was fitted with a DB-FFAP fused silica capillary column measuring 60 m × 320 µm × 0.5 µm (Agilent, Santa Clara, CA, USA). The injector temperature was 230 °C, and the column was operated in splitless mode. Helium was used as the carrier gas at a constant column flow of 1 mL/min, with a purity of 99.999% helium. The oven temperature was held at 40 °C for 3 min, subsequently increased to 230 °C at a rate of 4 °C/min, and held for 10 min. The electron ionization mode (EI) was used with 70 eV ionization energy. The ion source temperature was 230 °C, and the data were acquired in scan mode, covering a mass-to-charge ratio (*m*/*z*) range from 35 to 350. The concentration of each compound was determined by comparing its peak area to that of the internal standard.

## 3. Results and Discussion

### 3.1. Isolation and Morphological Characterization

**Figure 1:**
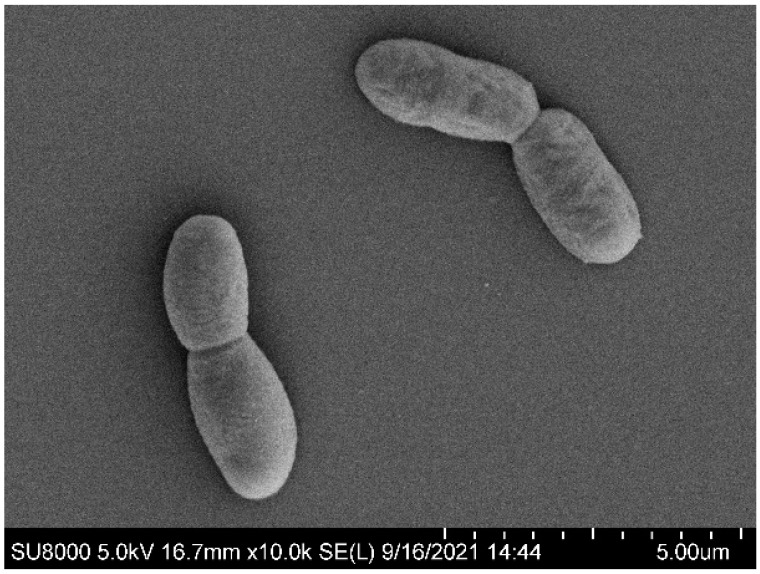
The cell morphology of WLY-B-L8^T^ observed by scanning electron microscopy (SEM) after incubation on PCA.

Colonies after 72 h of incubation on Tryptose Soya Agar (TSA) medium were circular, convex, and beige with a dry and opaque appearance, and an average diameter of 2 to 3 mm. The strain was a strictly aerobic, Gram-stain-positive, spore-forming bacterium, with rod-shaped cells of 0.7–1.3 μm in width and 1.6–3.4 μm in length, arranged singly or in pairs (Figure 1). WLY-B-L8^T^ was able to grow at 15–42 °C, with optimal growth at 40 °C. Strain WLY-B-L8^T^ was able to grow at pH 5.0–10.0, and the optimal pH for growth was 8.0–9.0. Optimal growth was achieved with 1% (*w*/*v*) NaCl, and no growth occurred with a concentration greater than 3.0% (*w*/*v*) NaCl for WLY-B-L8^T^.

### 3.2. Phylogenic Analysis

**Figure 2:**
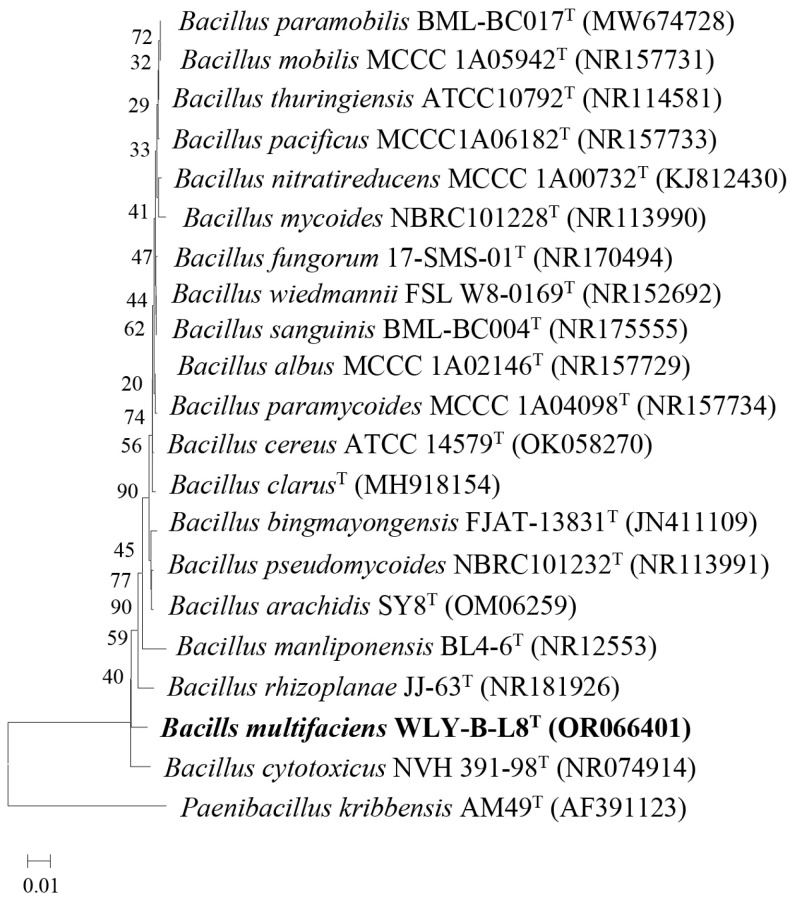
Phylogenetic tree based on 16S rRNA gene sequences, showing the relationships between strain WLY-B-L8^T^ and members of the genus *Bacillus*. The tree was reconstructed using the neighbor-joining method. Bootstrap values (>50%) based on 1000 replications are shown at branch nodes. The tree was rooted by *Paenibacillus kribbensis* AM49^T^ (AF391123) as the outgroup. Bar, 0.01 substitutions per nucleotide position.

The length of the determined sequence was 1398 bp. A BLAST (https://blast.ncbi.nlm.nih.gov/Blast.cgi?PROGRAM=blastn&PAGE_TYPE=BlastSearch&LINK_LOC=blasthome, accessed on 18 January 2025) search of the 16S rRNA gene sequence against the GenBank database showed that WLY-B-L8^T^ was related most closely to *Bacillus rhizoplanae* JJ-63 DSM 12442^T^ (98.71%), *Bacillus pseudomycoides* DSM 12442^T^ (98.21%), and *Bacillus cytotoxicus* NVH 391–98^T^ (98.14%). Phylogenetic analysis based on the 16S rRNA gene showed that strain WLY-B-L8^T^ formed a distinct branch with *Bacillus cytotoxicus* NVH 391–98^T^ in the neighbor-joining tree (Figure 2).

### 3.3. Physiological and Chemotaxonomic Characteristics

WLY-B-L8^T^ was found to be positive for gelatin hydrolysis, alkaline phosphatase, leucine arylamidase, chymotrypsin, catalase, acid phosphatase, *α*-glucosidase, *β*-glucosidase, esterase (C4), lipoid esterase (C8), naphthol AS-BI-phosphate hydrolase, *N*-acetylglucosaminase, nitrate reduction, and aesculin. but negative for citric acid utilization, valine arylamidase, cystine arylamidase, oxidase, arginine dihydrolase, the Voges–Proskauer (VP) test, glucose assimilation, trypsin, *α*-galactosidase, esterase (C14), *β*-galactosidase, *β*-glucuronidase, *α*-mannosidase, and *β*-fucosidase. This strain can also use *N*-acetylglucosamine, D-maltose, glucose, malic acid, and gelatin as its sole carbon sources. It differed from *Bacillus rhizoplanae JJ-63* DSM 12442^T^, *Bacillus pseudomycoides* DSM 12442^T^, and *Bacillus cytotoxicus* NVH 391–98^T^, in which the positive actions were related to acetoin production, D-glucose, D-fructose, D-maltose, cellobiose, glycogen, etc.; arginine dihydrolase, citrate utilization, acetoin production, D-ribose, arbutin, etc.; and acetoin production, gelatinase, D-ribose, D-mannose, arbutin, etc.; respectively. The detailed physiological and biochemical characteristics of WLY-B-L8^T^ and the reference strains are listed in Table 1. The strain WLY-B-L8^T^ was positive to starch hydrolysis and sucrose, which indicates it can secrete abundant enzymes to perform starch hydrolysis [23], while *Bacillus rhizoplanae JJ-63* DSM 12442^T^ was negative to starch hydrolysis or sucrose [27]. This may be because strain WLY-B-L8^T^ was isolated from *daqu*, which was the saccharification starter culture; that is, *daqu* contains rich glycoside hydrolase [28].

Our chemotaxonomic analysis results indicated that ribose, xylose, arabinose, mannose, glucose, and galactose constituted the major cell-wall sugars. MK-7 was the predominant respiratory quinone, which is typical of the large majority of members of the genus *Bacillus* [3]. Moreover, *meso*-diaminopimelic acid (*meso*-DAP) was the diagnostic amino acid. The main polar lipids of WLY-B-L8^T^ included diphosphatidylglycerol (DPG), phosphatidylglycerol (PG), and phosphatidylethanolamine (PE), which are characteristic lipids present in species of the genus *Bacillus* [3]. In addition, unidentified aminolipids (UAL 1–2), an unidentified aminophospholipid (UAPL), an unidentified aminoglycolipid (UAGL), and an unidentified lipid (UL) were also detected. The total fatty acid profile (Table 2) revealed that *iso*-C_15:0_ (23.0%) was the major component, and branched fatty acids accounted for 69% in strain WLY-B-L8^T^ (*iso*-C_15:0_ 23.0%, *iso*-C_17:0_ 8.9%, *iso*-C_13:0_ 7.9%, *iso*-C_17:1_ω5*c* 7.0%, *anteiso*-C_13:0_ 6.3%, *iso*-C_12:0_ 4.8%, C_18:3_ω6*c* 4.3%, C_16:0_ 3.9%, and *iso*-C_16:0_ 3.5%). This profile differs from that of *Bacillus rhizoplanae JJ-63* DSM 12442^T^, *Bacillus pseudomycoides* DSM 12442^T^, and *Bacillus cytotoxicus* NVH 391–98^T^, which predominantly comprised *iso*-C_15:0_ (19.9%), *iso*-C_17:0_ (16.9%), and *anteiso*-C_15:0_ (10.6%); *iso*-C_15:0_ (13.3%), *iso*-C_13:0_ (12.6%), and C_16:1_ω6*c* (12.3%); *iso*-C_15:0_ (38.6%), summed feature 3 (11.5%), and *iso*-C_17:0_ (11.4%); respectively. It can be seen from the fatty acid results that the first class fatty acid of the strain WLY-B-L8^T^ and the most closely related bacteria is *iso*-C_15:0_. The results are consistent with previous reports, in which *iso*-C_15:0_, *anteiso*-C_15:0_, *iso*-C_16:0_, *iso*-C_17:0_, and *anteiso*-C_17:0_ represent the major fatty acids typically found in *Bacillus* species [29]. The differences between the three strains indicate that novel strain WLY-B-L8^T^ likely represents a separate species in the genus *Bacillus*.

### 3.4. Genomic Features

The draft genome of WLY-B-L8^T^ consisted of 93 contigs and 4808568 bp (N50, 89695; G+C content, 35.97 mol%; accession number, JAVBXD000000000). The genome sequence of WLY-B-L8^T^ was 4.8 Mb in size. The G+C content of the type strain was determined as 35.97 mol%; in comparison, those of *B. rhizoplanae* JJ-63^T^, *B. pseudomycoides* DSM 12442^T^, and *B*. *cytotoxicus* NVH 391–98^T^ were 36.39, 35.37, and 35.87 mol%, respectively [27]. Gene prediction and annotation led to 4781 coding sequences. The genome sequence-based digital DNA–DNA hybridization (dDDH) values between *Bacillus rhizoplanae JJ-63*^T^, *Bacillus pseudomycoides* DSM 12442^T^, and *Bacillus cytotoxicus* NVH 391–98^T^ were 36.70%, 26.10, and 23.90%, respectively. These values are below the threshold value for the 70% dDDH species boundary [30], demonstrating that WLY-B-L8^T^ does not represent any of these related species. In addition, the average nucleotide identity (ANI) values between strain WLY-B-L8^T^ and the three aforementioned type strains were 88.24%, 80.57, and 78.70%. The average amino identity (AAI) values between them are 89.84%, 79.51%, and 80.41%. All of the ANI and AAI values are below the 95–96% cut-off values [31], indicating that WLY-B-L8^T^ represents a novel species of the genus *Bacillus*.

**Figure 3:**
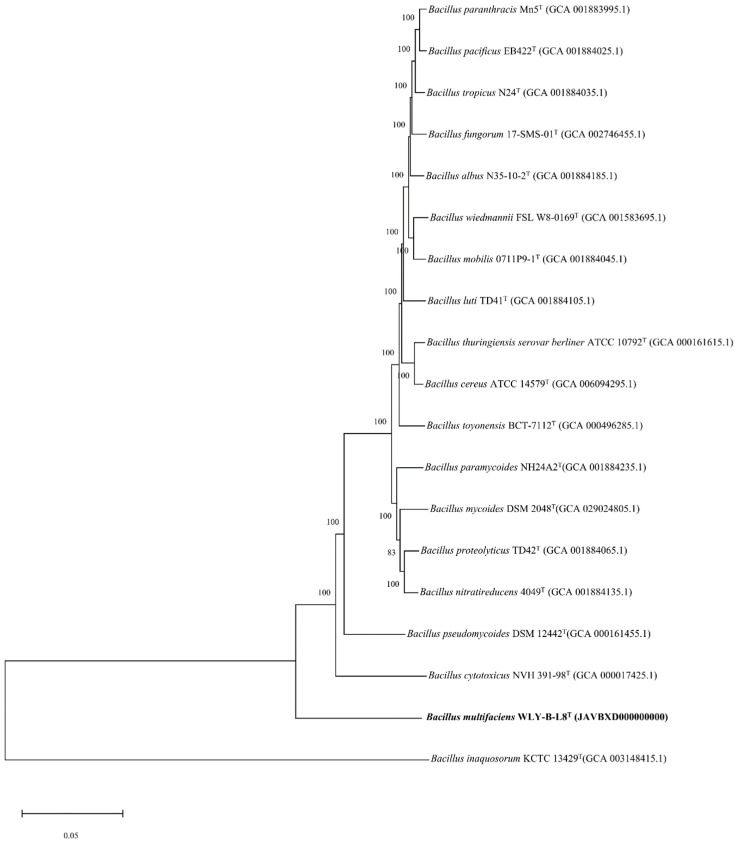
Phylogenomic tree based on three core marker proteins showing the phylogenetic position of WLY-B-L8^T^ among type strains of species of the genus *Bacillus*. Bar, 0.05 substitutions per nucleotide position.

The evolutionary relationships among bacteria can be studied by using orthologous genes of the core genome. The inference of the corresponding phylogenetic tree offers deeper insights. Hence, a phylogenomic analysis was conducted based on the 1041 core marker genes. The phylogenetic tree (Figure 3) also demonstrated a clear clustering of WLY-B-L8^T^ with the *Bacillus* sp.

**Figure 4:**
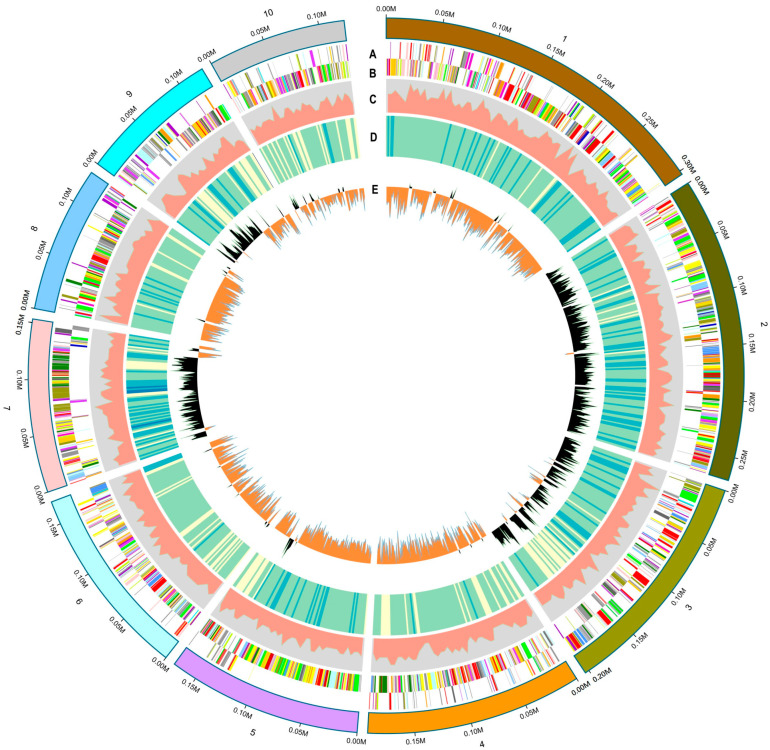
Graphical circular map of the genome from strain WLY-B-L8^T^. From the outside to center: cds (the nucleotide sequence for predicting genes), gene density, GC ratio, and GC skew (A: CDS+, B: CDS−, C: Gene density, D: GC ratio, E: GC skew).

The strain WLY-B-L8^T^ was predicted and contained 4781 coding sequences. Gene function analysis using KEGG showed that the majority of the genes were involved in global and overview maps (693 genes), amino acid metabolism (266 genes), carbohydrate metabolism (222 genes), the metabolism of cofactors and vitamins (162 genes), energy metabolism (143 genes), nucleotide metabolism (86 genes), and lipid metabolism (76 genes). Among the COG-recognized genes, 256 genes were related to an unknown function. The highest COG classification annotated to metabolism was related to amino acid transport and metabolism (347 genes), carbohydrate transport and metabolism (208 genes), coenzyme transport and metabolism (201 genes), inorganic ion transport and metabolism (187 genes), energy production and conversion (186 genes), and lipid transport and metabolism (151 genes). Figure 4 illustrates a graphical circular map of the genome from strain WLY-B-L8^T^.

### 3.5. Protease Enzyme Production Analyses

**Figure 5:**
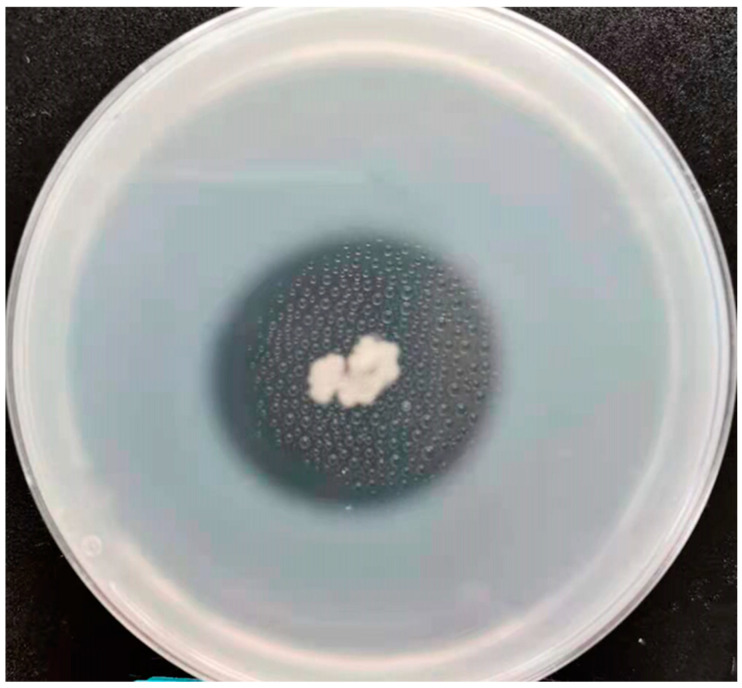
Plate diagram of proteinase production of strain WLY-B-L8^T^ (Created by authors).

The genus *Bacillus* is widely recognized as a crucial source of proteases and is distinguished by its significant production of protease enzymes [32]. *Bacillus* species play a pivotal role in shaping the distinctive saucy aroma of Chinese baijiu. These bacteria secrete a wide range of highly active enzymes, particularly proteases. This enzymatic activity is crucial for hydrolyzing fermentation substrates such as proteins, not only supporting their growth but also enhancing flavor compound production [33]. Proteases therefore play a crucial role in the production of baijiu. The qualitative determination results of protease activity show that this strain exhibits high protease activity, forming clear zones around the colonies on the aforementioned plates, with diameters measuring 2.1–2.2 cm (Figure 5). According to the light absorption value of the reaction between tyrosine and folinol, the standard curve was established. The protease activity of the strain was measured by calculating the amount of tyrosine produced by the reaction between the strain WLY-B-L8^T^ and tyrosine. The tyrosine absorbance (OD_660_) of the standard curve ranged from 0 to 0.8137, and the tyrosine concentration ranged from 0 to 0.10 g/L. The absorbance values for the three parallel measurements of the strain’s crude enzyme solution were 0.3316, 0.3651, and 0.3325, with an average value of 0.3431. The final protease activity of the new *Bacillus* strain WLY-B-L8^T^ was calculated to be 119.38 ± 7.44 U/mL. We found that *Bacillus subtilis*, isolated from species inhabiting hydrothermal vents, can produce protease using malt extract with a specific activity of 55.125 U/mg [34]. *Bacillus* has been reported to produce protease, which hydrolyzes macromolecules such as protein and promotes the formation of baijiu flavor [8]. However, in the literature, there are no reports of proteinase production in the three bacteria *Bacillus rhizoplanae* JJ-63 DSM 12442^T^, *Bacillus pseudomycoides* DSM 12442^T^, or *Bacillus cytotoxicus* NVH 391–98^T^ [27], which are the most closely related bacteria to the strain WLY-B-L8^T^, indicating that the microorganisms in *baobaoqu* have the ability to produce strong proteinase.

### 3.6. Flavor Compound Analyses

**Figure 6:**
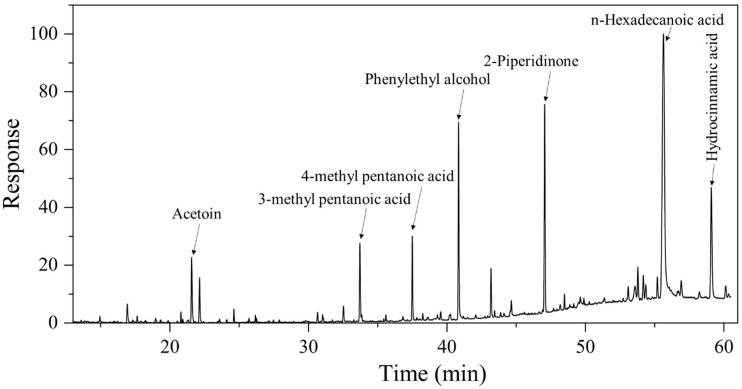
Chromatogram of flavor-producing substances of strain WLY-B-L8^T^.

Following the culture of WLY-B-L8^T^ in raw wheat for a period of 3 days, our results showed that 2-pipendinone (21.95 mg/L), phenylethyl alcohol (19.08 mg/L), hydrocinnamic acid (18.60 mg/L), and acetoin (7.58 mg/L) were the major volatile flavor compounds of WLY-B-L8^T^ (Table 3, Figure 6). However, they were barely detected in the blank medium. Piperidone compounds and their derivatives possess specific bioactive properties, including anticancer activity and the ability to treat central nervous system disorders [35,36]. In baijiu fermentation, microorganisms metabolize substrates such as proteins, fats, and carbohydrates. Carbohydrates are hydrolyzed into the key metabolic intermediate pyruvate through glycolysis and the tricarboxylic acid cycle. Pyruvate, generated via glycolysis, is then converted to ethanol by the enzymes pyruvate decarboxylase and alcohol dehydrogenase. Proteases hydrolyze soybean proteins into amino acids, including L-phenylalanine. These amino acids are further metabolized by amino acid transaminase into 3-phenylpyruvic acid, which is ultimately converted into phenethyl alcohol [8]. Phenylethyl alcohol is a derivative of aromatic hydrocarbons with a rose-like aroma and is an important active ingredient with antioxidant properties [37]. Hydrocinnamic acid can be used as a fixative in fragrances, possesses a mildly sweet aroma, and is an important flavor compound [38]. At present, however, there are no reports of these flavor compounds being produced in the three bacteria *Bacillus rhizoplanae* JJ-63 DSM 12442^T^, *Bacillus pseudomycoides* DSM 12442^T^, and *Bacillus cytotoxicus* NVH 391–98^T^ [27], which are the most closely related bacteria to the strain WLY-B-L8^T^, indicating that the microorganisms in *baobaoqu* have the ability to produce more flavor compounds. The strain WLY-B-L8^T^ can be widely applied in the brewing of strong-flavor baijiu, thereby enhancing the bodily quality of the baijiu.

### 3.7. Description of Bacillus multifaciens sp. nov.

This strain has been designated *Bacillus multifaciens* (mul.ti.fa’ci.ens. L. masc. adj. *multus*, many; L. pres. part. *faciens*, making; N.L. part. adj. *multifaciens*, making many compounds).

The strain is an aerobic, Gram-stain-positive, spore-forming bacterium, with rod-shaped cells 1.2–1.9 μm in width and 1.7–4.8 μm in length, arranged singly or in pairs. After 72 h of incubation, the colonies on TSA are circular, convex, and beige with a dry and opaque appearance and an average diameter of 2 to 3 mm. Growth occurs at 20–42 °C (optimum 40 °C), pH 5.0–10.0 (optimum pH 8.0–9.0), and NaCl concentrations of 0–2% (optimum 1%). Gelatin hydrolysis, alkaline phosphatase, leucine arylamidase, chymotrypsin, catalase, acid phosphatase, *α*-glucosidase, *β*-glucosidase, esterase (C4), lipoid esterase (C8), naphthol AS-BI-phosphate hydrolase, *N*-acetylglucosaminase, nitrate reduction, aesculin, *N*-acetylglucosamine, D-maltose, glucose, malic acid, and gelatin are utilized as its carbon sources. MK-7 is the predominant menaquinone and the main fatty acids are *iso*-C_15:0_, *iso*-C_17:0_, *iso*-C_13:0_, *iso*-C_17:1_ω5*c*, and *anteiso*-C_13:0_. The strain can produce protease (119.38 ± 7.44 U/mL) and volatile flavor components when cultured on raw wheat, such as 2-pipendinone (21.95 mg/L), phenylethyl alcohol (19.08 mg/L), hydrocinnamic acid (18.60 mg/L), and acetoin (7.58 mg/L).

The type strain is *Bacillus multifaciens* WLY-B-L8^T^ (CICC 25210^T^ = JCM 36284^T^), isolated from the air at the Wuliangye *baobaoqu* production facility in Yibin city in Sichuan province, China. The DNA G+C content of strain WLY-B-L8^T^ was 35.97 mol%. The GenBank accession number for the 16S rRNA gene sequence of strain WLY-B-L8^T^ is OR066401 and the DDBJ/ENA/GenBank accession number for the draft genome is JAVBXD000000000.

## 4. Conclusions

In this study, we present a detailed characterization and taxonomic classification of *Bacillus multifaciens* sp. nov., a recently identified aerobic bacterium isolated from the air of the *qu*-starter of Chinese Wuliangye baijiu. The 16S rRNA gene sequence analysis and genomic features, including the ANI, AAI, and dDDH values, distinctly place WLY-B-L8^T^ within the *Bacillus* genus but as a separate lineage. The physiological and biochemical characteristics of WLY-B-L8^T^ were also found to be distinct from the other species within the genus, highlighting its novelty. Interestingly, WLY-B-L8^T^ is able to use the raw wheat of Wuliangye *baobaoqu* to produce volatile flavor compounds including 2-pipendinone (22.0 mg/L), phenylethyl alcohol (19.1 mg/L), hydrocinnamic acid (18.6 mg/L), acetoin (7.6 mg/L), and so forth. These substances are important volatile flavor components of Wuliangye baijiu. The findings of this study extend our knowledge of the *Bacillus* species and their ecological roles in the distillery environment.

## Figures and Tables

**Table 1:** Phenotypic characteristics of strain WLY-B-L8^T^ and closely related phylogenetic neighbors in the genus *Bacillus*.

Characteristics	1	2	3	4
Cell length (μm)	1.6–3.4	3.0–4.0	3.0–5.0	NR
Cell width (μm)	0.7–1.3	>1.0	>1.0	>1.0
Oxidase/catalase	−/+	+/+	+/+	+/+
Rhizoid colony	−	−	−	−
Growth temperature range (°C)	20–40	10–36	10–45	20–50
Optimal growth temperature (°C)	40	30	30(37)	30–37
Growth NaCl range (%)	0–2	0–3	0–4	NR
Optimal growth NaCl (%)	1	1.0–2.0	0	NR
Growth pH range	5.0–10.0	5.5–10.5	5.0–9.5	NR
Optimal growth pH	8.0–9.0	7.0–8.0	6.0	NR
Starch hydrolysis	+	−	+	−
API 20E results				
*β*-Galacosidase	−	−	−	−
Arginine dihydrolase	−	−	+	−
Citrate utilization	−	−	+	−
Urease	−	−	−	−
Acetoin production (Voges−Proskauer)	−	+	+	+^w^
Gelatinase	+	−	+	+
Fermentation/oxidation of glucose	−	−	−	−
API 50CH results				
D-Ribose	−	−	+	+
D-Xylose	−	−	−	−
D-Galactose	−	−	−	−
D-Glucose	+	+	+	+
D-Fructose	+	+	+	+
D-Mannose	−	−	−	+
D-maltose	+	+	+	+
*N*-Acetylglucosamine	+	+	+	+
Arbutin	−	−	+	+
Aesculin ferric citrate	−	−	+	+
Salicin	+^w^	−	+	+
Cellobiose	−	+	+	+
Sucrose	+	−	+	−
Trehalose	−	−	+	−
Starch	+	+	+	−
Glycogen	−	+	+	−
Potassium gluconate	−	−	−	−
DNA G+C content (mol%)	35.97	36.39	35.37	35.87

Strains: 1, WLY-B-L8^T^; 2, *Bacillus rhizoplanae JJ-63* DSM 12442^T^; 3, *Bacillus pseudomycoides* DSM 12442^T^; and 4, *Bacillus cytotoxicus* NVH 391–98^T^. Data for strains 2–4 from [24]. In the API 20E tests, all strains were negative for lysine decarboxylase, ornithine decarboxylase, H_2_S production, trypophan deaminase indole production, mannitol, inositol, sorbitol, rhamnose, sucrose, melibiose, amygdalin, and arabinose. In the API 50 CHB tests, all strains were negative for erythritol, D-arabinose, L-arabinose, L-xylose, D-adonitol, methyl *β*-D-xylopyranoside, L-sorbose, L-rhamnose, dulcitol, inositol, mannitol, sorbitol, methyl α-D-mannopyranoside, D-lactose, D-melibiose, inulin, D-melezitose, D-raffinose, xylitol, D-gentiobiose, D-luxose, D-tagatose, D-fucose, L-fucose, D-arabitol, L-arabitol, potassium 2-ketogluconate, and potassium 5-ketogluconate. +, positive; −, negative; +^w^, weakly positive; NR, not reported, the parameter was not tested.

**Table 2:** Comparative FAME profiles of strain WLY-B-L8^T^ and closely related phylogenetic neighbors in the genus *Bacillus*.

Fatty Acid	1	2	3	4
C_12:0_	-	ND	1.4	ND
C_14:0_	1.1	3.2	3.2	3.2
C_16:0_	3.9	8.6	9.0	5.6
C_15:1_ω5*c*	1.9	ND	ND	ND
C_16:1_ω6*c*	-	8.4	12.3	ND
C_16:0_N alcohol	1.2	ND	ND	ND
C_17:0_	1.3	ND	ND	ND
C_18:0_	1.7	ND	TR	ND
C_18:3_ω6*c*	4.3	ND	ND	ND
*iso*-C_12:0_	4.8	ND	8.7	ND
*iso*-C_13:0_	7.9	8.1	12.6	7.1
*iso*-C_14:0_	1.3	2.9	5.5	2.3
*iso*-C_15:0_	23.0	19.9	13.3	38.6
*iso*-C_16:0_	3.5	6.9	8.3	5.1
*iso*-C_16:1_*cis*10	-	2.9	-	-
*iso*-C_16:1_ω5	-	ND	2.9	ND
*iso*-C_17:0_	8.9	16.9	7.0	11.4
*iso*-C_17:1_ω5*c*	7.0	ND	ND	ND
*iso*-C_17:1_ω6	-	ND	2.3	ND
*anteiso*-C_13:0_	6.3	3.5	4.9	ND
*anteiso*-C_15:0_	2.9	10.6	3.6	3.1
*anteiso*-C_17:0_	1.4	3.3	1.6	ND
*anteiso*-C_17:1_*a*	2.1	ND	ND	ND
*anteiso*-C_17:1_*cis*5	-	4.6	-	-
*anteiso*-C_17:1_ω6	-	ND	1.1	ND
Summed feature 2	2.6	-	ND	1.5
Summed feature 3	6.7	-	ND	11.5

Values are percentages of the total fatty acids. Only fatty acids showing relative amounts > 1% are given. Strains: 1, WLY-B-L8^T^; 2, *Bacillus rhizoplanae JJ-63* DSM 12442^T^; 3, *Bacillus pseudomycoides* DSM 12442^T^; and 4, *Bacillus cytotoxicus* NVH 391–98^T^. Data for strains 2–4 from [27]. ND, no data; TR, trace.

**Table 3:** Concentration of volatile compounds in cultivated powder by WLY-B-L8^T^.

Volatile Compounds	Concentration (mg/L)
Acetoin	7.58 ± 0.11
3-methyl pentanoic acid	8.80 ± 0.24
4-methyl pentanoic acid	7.42 ± 0.19
Phenylethyl alcohol	19.08 ± 0.82
2-Piperidinone	21.95 ± 1.56
*n*-Hexadecanoic acid	78.25 ± 2.19
Hydrocinnamic acid	18.60 ± 0.53

## Data Availability

The original contributions presented in this study are included in the article; further inquiries can be directed to the corresponding authors.

## Abbreviations

dDDH: digital DNA–DNA hybridization; PCR: Polymerase Chain Reaction; GGDC: Genome-to-Genome Distance Calculation; PCA: Plate Count Agar; TSA: Tryptose Soya Agar; ANI: average nucleotide identity; AAI: average amino identity; FAME: fatty acid methyl ester; HS-SPME: headspace solid phase microextraction; GC-MS: gas chromatography mass spectrometry.
